# Learning Crystallographic Disorder: Bridging Prediction and Experiment in Materials Discovery

**DOI:** 10.1002/adma.202514226

**Published:** 2025-10-23

**Authors:** Konstantin S. Jakob, Aron Walsh, Karsten Reuter, Johannes T. Margraf

**Affiliations:** ^1^ Theory Department Fritz Haber Institute of the Max Planck Society Faradayweg 4‐6 14195 Berlin Germany; ^2^ Department of Materials Imperial College London London SW7 2AZ UK; ^3^ Chair of Physical Chemistry V: Theory and Machine Learning University of Bayreuth Bavarian Center for Battery Technology (BayBatt) Weiherstraße 26 95448 Bayreuth Germany

**Keywords:** disorder, ICSD, machine learning, materials discovery

## Abstract

Recent computational materials discovery efforts have led to an enormous number of predictions of previously unknown, potentially stable inorganic, crystalline compounds. In particular, both high‐throughput screenings and generative models have benefited tremendously from recent advances in computational resources and available data. However, these efforts are currently limited to predicting pristine crystalline materials. As a consequence, many of these predictions cannot be realized in experiments, where kinetic effects, defects, and crystallographic disorder can be crucial. To address this shortcoming, the current work aims to introduce disorder into computational materials discovery with machine learning (ML) based classification models. Trained on the inorganic crystal structure database (ICSD), these classifiers capture the chemical trends of crystallographic disorder and estimate the prevalence of disorder in computational databases produced by the Materials Project or Graph Networks for Materials Science (GNoME) initiatives. This opens the door toward disorder‐aware computational materials discovery workflows, bridging the gap between prediction and experiment.

## Graphical abstract

Machine learning based computational materials discovery workflows have recently proposed thousands of potentially stable crystalline materials. However, the experimental realization of these predictions is often challenging because the models assume perfectly ordered structures. This work introduces machine learning classification models that predict how likely a novel material is to display crystallographic disorder, opening the door toward disorder‐aware materials discovery workflows.

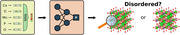

## 1 Introduction

In recent years, the field of materials discovery has profited immensely from the development of large‐scale databases and novel machine learning (ML) tools.^[^ ^1^, ^2^, ^3^, ^4^, ^5^ ^]^ This advance has catalyzed the development of a variety of discovery workflows, ranging from the computational screening of large databases to structure generation using element substitution methods,^[^ ^1^, ^6^ ^]^ random structure search,^[^ ^4^, ^7^ ^]^ or (deep) generative models. The latter includes a staggering diversity of ML architectures such as variational autoencoders,^[^ ^8^, ^9^ ^]^ generative adversarial networks,^[^ ^10^, ^11^ ^]^ diffusion models,^[^ ^12^, ^13^, ^14^, ^15^ ^]^ autoregressive transformers,^[^ ^16^, ^17^, ^18^ ^]^ or evolutionary algorithms.^[^ ^19^, ^20^ ^]^ In addition, the emergence of generally applicable machine learning interatomic potentials (MLIPs) such as M3GNet,^[^ ^21^ ^]^ MACE‐MP‐0,^[^ ^22^ ^]^ or SevenNet‐0,^[^ ^23^ ^]^ has further accelerated materials discovery efforts drastically compared to purely first‐principles based workflows.^[^ ^4^, ^6^ ^]^

At their core, most computational materials discovery efforts at least indirectly rely on density functional theory (DFT) based relative energies of ordered crystal structures at 0 K, to estimate the thermodynamic stability of a proposed material. This approach gives rise to the so‐called convex hull of thermodynamically stable compositions.^[^ ^24^ ^]^ As it allows predicting a materials stability (relative to other known phases) from a single DFT relaxation, this concept is convenient from a computational point of view. Unfortunately, it is well known that many materials that lie on the DFT convex hull are not readily synthesizable, while some systems that lie above the hull occur naturally.^[^ ^25^, ^26^, ^27^ ^]^ This discrepancy can have numerous causes, from errors in the DFT potential energy surface,^[^ ^25^ ^]^ to kinetic factors in crystallization,^[^ ^28^ ^]^ to effects of finite temperature and pressure.^[^ ^25^ ^]^ One particular aspect (related to finite temperature) that has come into view recently is crystallographic disorder, which we will focus on here.^[^ ^29^ ^]^

Disorder is a common feature of real materials, especially when going from 0 K to realistic conditions during synthesis, characterization, or operation.^[^ ^30^ ^]^ Disordered materials have gained considerable attention in literature, for instance in high‐performance, light‐weight solar cells,^[^ ^31^ ^]^ ferroelectrics,^[^ ^32^ ^]^ catalytic applications,^[^ ^33^, ^34^, ^35^, ^36^, ^37^ ^]^ and as small‐gap semiconductors.^[^ ^38^ ^]^ (Cation‐)disordered rock salt (DRX) materials, such as Li_1.211_Mo_0.467_Cr_0.3_O_2_ are investigated as promising cathode materials for Li‐ion batteries due to positive impacts on capacity, conductivity, and stability of the resulting batteries.^[^ ^39^, ^40^, ^41^ ^]^ Hence, crystallographic disorder in realistic materials is increasingly turning into a design parameter in applications, and there is a severe need for materials discovery workflows to include and recognize disordered materials.

The computational description of disordered materials remains challenging. It generally requires sampling an ensemble of configurations and evaluating their energy distribution to estimate the entropic contribution to the formation enthalpy.^[^ ^42^ ^]^ Furthermore, large supercells can be required to host the stoichiometric ratio of complex compositions and avoid finite size effects.^[^ ^42^ ^]^ Due to the large computational cost of DFT calculations, a brute force treatment of crystallographic disorder therefore becomes intractable even for small materials spaces. To overcome this limitation, methods like the virtual crystal approximation (VCA) can be employed,^[^ ^43^ ^]^ where disordered sites in a crystal are approximated by an averaged Hamiltonian. This approximation leads to a neglect of important short‐range correlations and the specific interactions that the disordered sites share, however. More sophisticated approaches focus on increasing the system size, for instance by generating large, disordered supercells and ensembles thereof to accurately take interactions of specific arrangements of disorder into account.^[^ ^44^, ^45^, ^46^ ^]^ For example, the alloy cluster expansion (CE) method as implemented in ^[^ ^47^ ^]^ expands the energy of a disordered systems by an ensemble of differently sized, disordered supercells and optimizes this expansion using Monte Carlo algorithms.^[^ ^46^ ^]^ These approaches interface well with the current developments of (general purpose) MLIPs, allowing to extend unit scale sizes drastically with reasonable computational cost.^[^ ^45^ ^]^ However, the computational demands of such approaches remain unfeasible for large‐scale discovery efforts. As a result, crystallographic disorder remains largely neglected in this context.

In this study, we first address the question about the relevance of crystallographic disorder for materials discovery. To this end, we analyze chemical trends of crystallographic disorder in the Inorganic Crystal Structure Database (ICSD), a diverse data set containing ca. 220 000 ordered and disordered experimental crystal structures.^[^ ^48^ ^]^ Furthermore, we exploit these patterns and trends by developing ML classification models to recognize compositions that are prone to displaying disorder. Finally, we reevaluate predictions of large‐scale materials discovery efforts on the examples of the Materials Project (MP) and GNoME in the context of disorder and attempt to quantify the fraction of disordered materials in such databases.

## 2 Results

### 2.1 Disorder in the ICSD

To develop an intuition about the prevalence for disorder in real crystalline materials, we analyze the ICSD. The ICSD is currently the largest database of fully resolved inorganic crystal structures determined from experiment, giving rise to a chemically diverse set of materials with known disorder. Importantly, the ICSD is also the basis for the Materials Project (MP) database, a widely used computational database and starting point for many materials discovery efforts. At the time of our data collection, the ICSD contained 219 719 crystal structures of 138 426 unique compositions in the form of Crystallographic Information Files (cif). These files contain the structure itself (i.e., atomic positions, site occupancies, and crystal symmetry information), as well as metadata such as synthesis and measurement conditions. Of these 220 000 entries, 102 637 (47%) are disordered. From this number, it is clear that disorder is an important phenomenon that must be considered in materials discovery.

**Figure 1:**
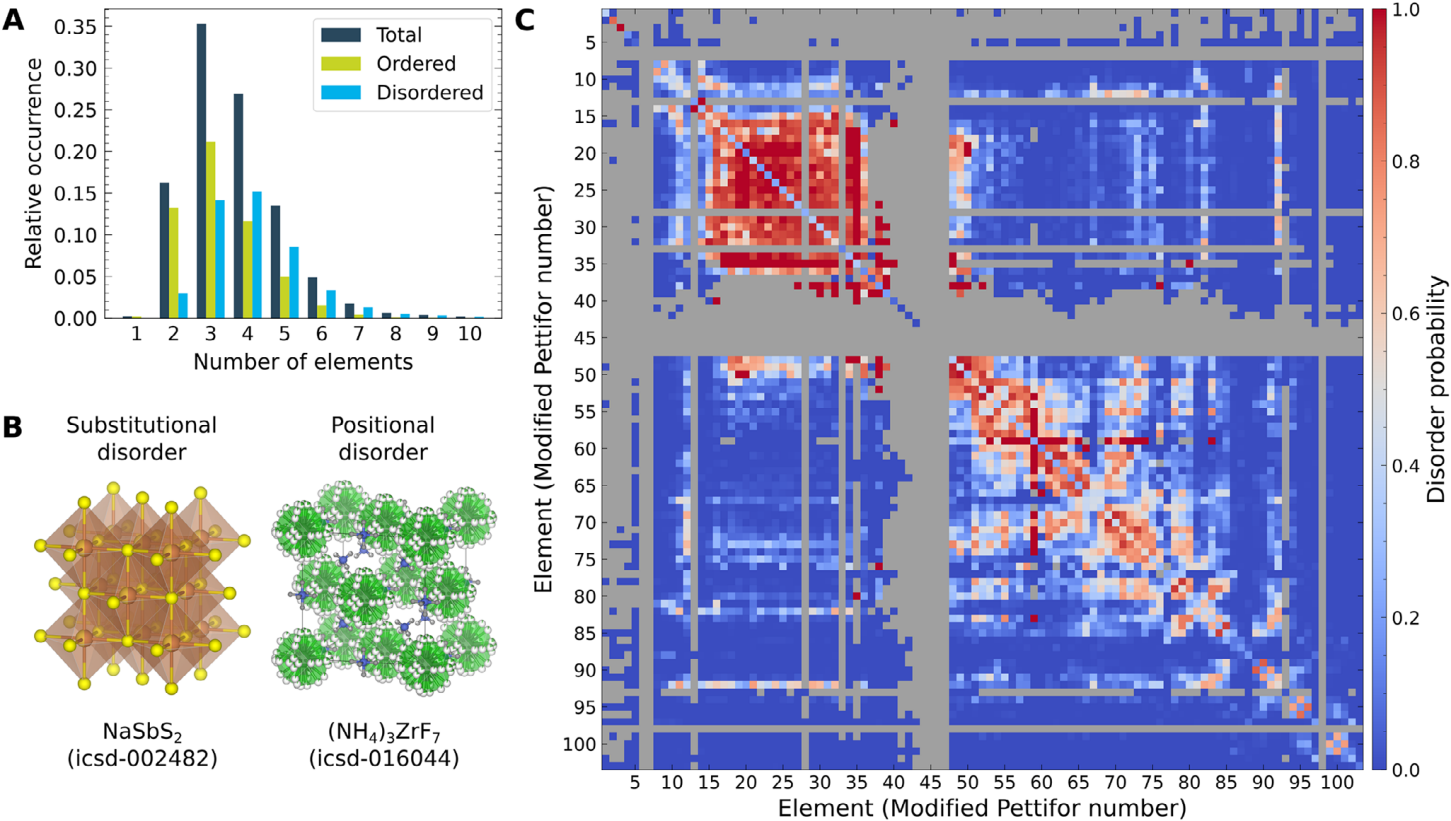
Visualization of disordered materials in the Inorganic Crystal Structure Database (ICSD).^[^ ^48^ ^]^ A) Distribution of materials as a function of the number of elements for the entire ICSD (dark blue), ordered materials only (light green), and disordered materials only (light blue). Most ordered materials are ternary compounds, most disordered materials are quaternary compounds. The fraction of ordered materials decreases with the number of elements. B) Examples for disorder in the ICSD. Materials can display substitutional disorder (left, NaSbS_2_), where two or more elements share a crystallographic site, and positional disorder (right, (NH_4_)_3_ZrF_7_), where elements occupy a site only partially, e.g., due to rotation of a polyhedron. Hydrogens are shown in dark grey to differentiate them from vacancies shown in white. We find more substitutionally (73%) than positionally (46%) disordered materials in the ICSD. C) Probability of disorder for elements and element pairs in materials of the ICSD. Shown are the probability for an element to exhibit positional disorder (diagonal), and to share a crystallographic site with another element in substitutional disorder (off‐diagonal). Elements are arranged by modified Pettifor number.^[^ ^49^ ^]^ High probabilities for disorder are shown in blue, while low probabilities are shown in red. The grey background indicates elements and element combinations that do not occur in the ICSD.

In **Figure** **1**A, we show the distribution of materials in the ICSD as a function of the number of elements in the composition for all (dark blue), ordered (green), and disordered (light blue) materials. The total distribution of materials peaks for ternary compounds, indicating that these materials are most frequent in the ICSD. Ordered materials follow this trend and contribute most to the distribution for ternaries and below. Disordered systems, however, are most prominent in the ICSD for quaternary compositions and dominate the distribution more and more with an increasing number of elements in the chemical system. This behavior is intuitive as more elements increase the likelihood for two elements sharing a crystallographic site, for instance due to a higher likelihood of hosting similar elements as well as a generally higher expected configurational entropy. At the same time, this shift between ordered and disordered distribution shows that ‐ especially when designing complex materials with many elements ‐ disorder can dominate the equilibrium crystal structures in the materials space.

Disordered systems in the ICSD can be further categorized into materials with positional disorder and materials with substitutional disorder. Positional disorder describes crystal structures in which a single species is statistically split across several mutually exclusive sites or partially occupies a single symmetry‐averaged site. Examples for positional disorder are thus rotational degrees of freedom, statistical tilting of coordination polyhedra, or the formation of interstitial defects within the crystal. Substitutional disorder describes statistical chemical mixing on one crystallographic site, where two or more different species share that site with fractional occupancies summing to unity. An example from the ICSD for either type is provided in Figure 1B. We find that 74 610 (73%) of the 102 637 disordered materials display substitutional disorder, while 46 996 (46%) show positional disorder (with 18 969 (18%) showing both forms of disorder). These numbers indicate that both forms of disorder are relevant in the ICSD, though substitutional disorder is more frequent.

To visualize the chemical origins behind this disorder, we determine a probability for two elements to share a crystallographic site in a disordered crystal over all entries of the ICSD. This probability can be obtained by counting the number of materials in which two elements share a crystallographic site, and then normalizing over the total occurrences of the specific element pair. For positional disorder, the same method can be applied, however, the element in question then shares a site with a vacancy. The probability matrix for arbitrary element pairs to occur in disordered structuresis shown for the full periodic table in Figure 1C. Here, the elements are arranged on both axes according to the modified Pettifor number *Z*_mP_,^[^ ^49^ ^]^ a one‐dimensional representation of the periodic table where chemically similar elements are close to each other. Probabilities of positional disorder are included on the diagonal of the matrix.

Analysis of this probability matrix reveals several chemical trends driving the prevalence of disorder. First, the highest probability of substitutional disorder is found close to the diagonal, i.e., substitutional disorder occurs mainly if two fairly similar elements are present in the same material. This raises the question, whether simple elemental properties such as electronegativities or atomic radii are sufficient to predict the presence of disorder. Using the disorder probabilities from Figure 1C, we find that similarity in terms of these descriptors is a necessary but not sufficient condition for the presence of disorder, highlighting the need for more sophisticated classification models (see Figure S4, Supporting Information). The trend toward element similarity is for example pronounced for rare earth metals (*Z*_mP_ 18–30), as indicated by a large red area in the top left corner of Figure 1C. This behavior matches chemical intuition, given that rare earth metals are often used as dopants or in stoichiometric mixtures.^[^ ^50^, ^51^, ^52^ ^]^ Second, we find that the probability for anions and cations to share a crystallographic site is virtually 0, as can be seen from the blue, horizontal bands on the bottom left of the matrix. Again, these probabilities are intuitive, as site mixing between anions and cations would violate local charge neutrality in the crystal. Finally, we find that carbon and nitrogen (*Z*_mP_ 86 and 87 respectively) rarely partake in substitutional disorder. We speculate that this behavior is related to their small ionic radius, that can rarely be matched by other anions.

### 2.2 Classification Models

The trends in Section 2.1 demonstrate that some compositions are more prone to crystallize in disordered structures than others. These findings motivate us to develop ML classifiers to distinguish ordered and disordered materials reliably. Such classifiers could serve as post‐processing tools in materials discovery to differentiate those materials for which the 0 K convex hull approximation is likely valid from those that are potentially disordered.

To this end, we first prepare a curated data set from the full ICSD. In particular, we exclude systems with positional disorder from further analysis. This decision is rooted in the fact that positional disorder frequently occurs in systems with non‐integer stoichiometries that can easily be separated from potentially ordered compositions. Furthermore, positional disorder is often caused by rotation of small molecular units (or solvent molecules) in a crystal structure, such as H_2_O, NH_4_^+^, or PO_4_^3 −^. These represent a special class of systems, which are not usually the main target of large‐scale materials discovery efforts. Finally, it is worth pointing out that the position of hydrogen atoms determined by X‐ray diffraction carries a large uncertainty regardless and that these are sometimes placed according to potential coordination environments, leading to additional uncertainty in terms of positional disorder. From the remaining systems, we consider only structures measured at normal temperature and pressure (NTP) conditions, i.e., 293 K and 101.325 kPa, to ensure a consistent and homogeneous data set. This curation is especially important since disorder is a function of the thermodynamic conditions. Further details about the data set and our curation are given in the Experimental Section and the Supporting Information (Figures S1–S3). The curated data set contains 118 684 crystal structures with 79 788 unique compositions. Of these crystal structures, 77 571 (65%) have no shared crystallographic sites and are considered as ordered, whereas 41 113 (35%) are disordered. For model training and validation, this set is randomly split into training, validation, and test sets with a ratio of 60%: 10%: 30%.

**Figure 2:**
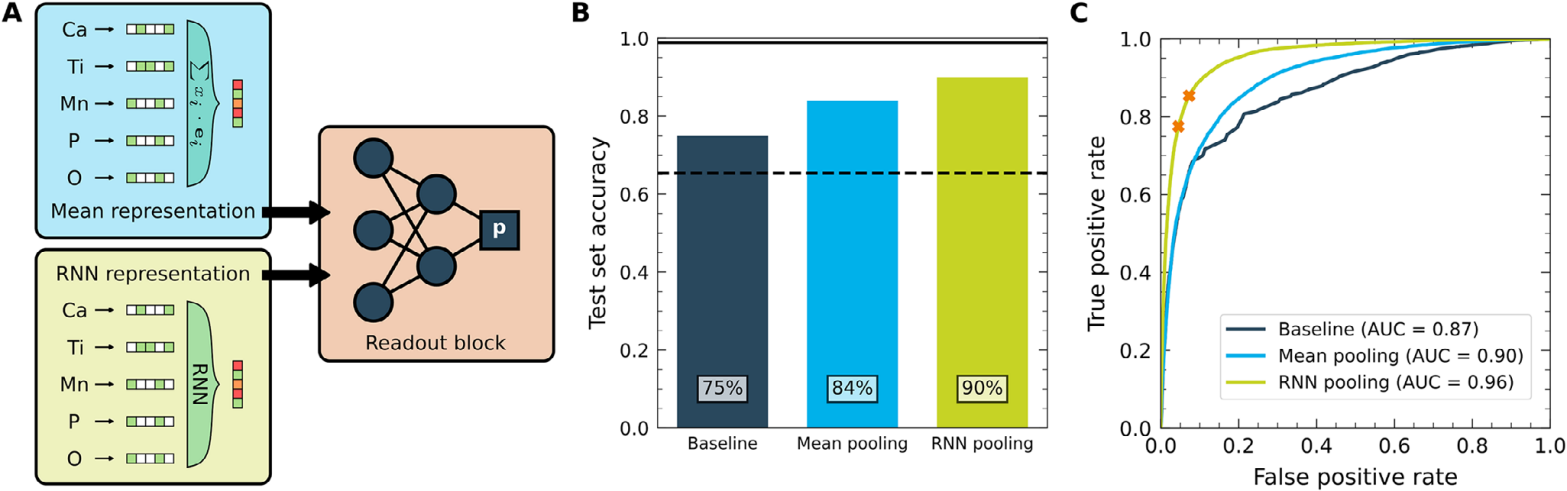
Classification models for disorder based on inorganic compositions. A) Model architectures of the two neural network classifiers. The models both consist of an embedding (blue/green background) and a readout (orange background) block. The mean pooling model (top, blue) uses compositionally pooled element embeddings, the recurrent neural network model (bottom, green) combines element embedding vectors sequentially using a long short‐term memory (LSTM) block.^[^ ^53^, ^54^ ^]^ B) Test set accuracy of the classifiers. The neural network classifiers with mean (light blue) and RNN pooled (light green) element embeddings show significantly higher accuracy than a trivial classifier that predicts only ordered labels (dashed line) and than a baseline using pair‐wise probabilities (dark blue) with probabilities derived from Figure 1. The maximum possible accuracy of 99% is indicated by the solid black line. C) Receiver operating characteristic for the baseline model (dark blue), the mean pooled embeddings (light blue), and the RNN model (light green). The RNN classifier has the highest area under the curve (ROC‐AUC score) of 0.96. The two models used in Section 2.3 are highlighted by the orange crosses.

The neural network (NN) classification models used in the following are based on pre‐trained element embeddings.^[^ ^53^ ^]^ These embeddings are vectorial representations of chemical elements that incorporate general information about the chemical nature of each element in a composition.^[^ ^53^, ^55^ ^]^ They can thus be understood as higher dimensional generalizations of the (modified) Pettifor scale. Specifically, we considered the Matscholar, Mat2Vec, and cgnf embeddings.^[^ ^56^, ^57^, ^58^ ^]^ To transform these element embeddings into representations of materials (or rather their compositions), they need to be pooled. Herein, we pursue two model architectures shown in **Figure** **2**A, consisting of a representation block and a readout block. In the first case, we choose a simple mean pooling of the element embeddings. In the second case, a trainable pooling layer based on a recurrent neural network (RNN) (specifically a long short‐term memory, LSTM, network) is used.^[^ ^54^ ^]^ This architecture enables a more sophisticated consideration of the context in which elements are combined, compared to mean pooling. More details about the models and optimal hyperparameters are presented in Classification Models Section.

In Figure 2B, the test set accuracy of these ML models is shown along with two baseline classifiers. On the one hand, a trivial classifier that simply labels all materials as ordered would achieve a performance of 65% accuracy. This performance is equivalent to an NN classifier suffering from mode collapse. On the other hand, a data‐driven classification baseline can be constructed from the matrix shown in Figure 1. To this end, we predict the probability of disorder based on the maximum likelihood of disorder for any element pair in a given composition. This baseline reaches a better accuracy of 75%, but shows a tendency to overestimate the prevalence of disorder (see confusion matrices in Figure S6, Supporting Information).

Both ML models reach a higher accuracy on the test set, with 84% in the case of the mean‐pooled NN and 90% in the case of the RNN. This improvement demonstrates that both models profit from knowledge about all elements in the composition as opposed to the data‐driven baseline, where only one specific element pair contributes to the decision. On the other hand, the stronger performance of the RNN classifier compared to mean pooling shows that the trainable pooling layer benefits from being able to assign composition‐dependent weights to individual elements. The composition‐based RNN model can be further improved slightly by incorporating CrystalNN site fingerprints,^[^ ^59^ ^]^ an exclusively geometric descriptor for the coordination environment of each crystallographic site (see Supporting Information for details). This indicates that higher accuracy can be achieved with structure‐aware models, though the applicability of these models is limited to cases where the crystal structure is known. It should also be noted that the pre‐trained element embedding vectors are used without further refinement, leaving room for further optimization of both classifiers.

To further analyze the performance, receiver operating characteristics (ROC)^[^ ^60^ ^]^ of the baseline model (dark blue), the mean pooling model (light blue), and the RNN model (light green) are shown in Figure 2C. The ROC curve shows the true positive and false positive rates for different classification thresholds (i.e. different classifier probabilities above which a composition is predicted to be disordered), with a larger area under the curve (ROC‐AUC score) being desirable. The RNN model has a ROC‐AUC of 0.96 and significantly outperforms both the baseline (ROC‐AUC 0.87) and the mean pooling model (ROC‐AUC 0.90). The ROC also allows for calibrating the threshold of classification, according to the desired false positive rate. Varying the classification threshold thus also provides some degree of uncertainty estimation of our predictions. In the following, we use two distinct thresholds for the RNN model, a balanced version with a threshold of 50%, and a conservative one with a threshold of 70%. The latter was chosen such that the false positive rate on the ICSD test set is just below 5%, with the drawback of a lower true positive rate. This choice gives some benefit of the doubt to computational predictions. To further mitigate the risk of over‐ or underfitting, more complex techniques such as committee approaches for uncertainty estimation or additional validation of the predictions with Monte‐Carlo sampling techniques could be applied.

Overall, these results show that substitutional disorder in inorganic crystals can be predicted with reasonable accuracy from the composition of the material alone. Materials discovery workflows can profit from this classification in two ways: First, the classifiers can serve as a post‐processing tool of newly proposed materials, serving as an indicator of possible competing disordered phases. In other words, the classification models can effectively distinguish those materials, where a computational structure prediction based on first‐principles methods is sufficient, from those, where more sophisticated modeling of phase equilibria (e.g., with Monte–Carlo simulations) is required. Second, the classifiers can also guide discovery efforts into domains of the chemical space where substitutional disorder is unlikely and where the 0 K picture of the convex hull is often sufficient, in particular if disorder is not desired in the application of the material.

### 2.3 Disorder in Computational Materials Databases

In recent years, computational materials discovery efforts have suggested a large number of novel, potentially stable crystal structures. Two prominent examples of this are the MP and GNoME databases.^[^ ^1^, ^4^ ^]^ The MP database consists of over 155 000 structures corresponding to ca. 105 000 unique compositions.^[^ ^1^ ^]^ Initially, the MP was derived from an ordered subset of the ICSD, but it has been subsequently expanded with computational predictions, e.g. based on element substitution or other materials discovery approaches. In contrast, the GNoME database consists of computational predictions that have been produced in a high‐throughput, active learning effort. As part of this work, ca. 381 000 new configurations which form a new convex hull below the MP reported systems have been reported as new discoveries. Given the above mentioned challenges in experimentally validating such computational predictions (and the fact that disordered systems are a blind spot in the MP), we now apply the RNN classifier to both databases.

In this analysis, we predict that 39–47% of the configurations in the MP database are likely to display substitutional disorder (excluding the overlap with the ICSD), using the conservative and balanced classification thresholds, respectively. This rate is slightly above the fraction of disordered materials in the curated ICSD (35%, see above). Thus, the MP has accumulated a sizable fraction of potentially disordered systems through computational predictions, given that the MP database originated from an ordered subset of the ICSD. In the GNoME set, the classifier predicts a much higher fraction of disordered materials, ranging from 80% to 84%. On one hand, this finding can be attributed to the fact that the explored materials space is significantly larger compared to the ICSD or MP, with a larger fraction of compositions with 4 or more elements. Such compositions, as illustrated in Section 2.1, have a proclivity for disorder. Furthermore, the materials predicted by the GNoME project intentionally focus on compositions that are not included in other computational databases, such as the MP or the Open Quantum Materials Database (OQMD).^[^ ^61^, ^62^ ^]^ Given that these databases are based on ordered ICSD subsets, their exclusion can lead to a bias toward potentially disordered materials in GNoME.

**Figure 3:**
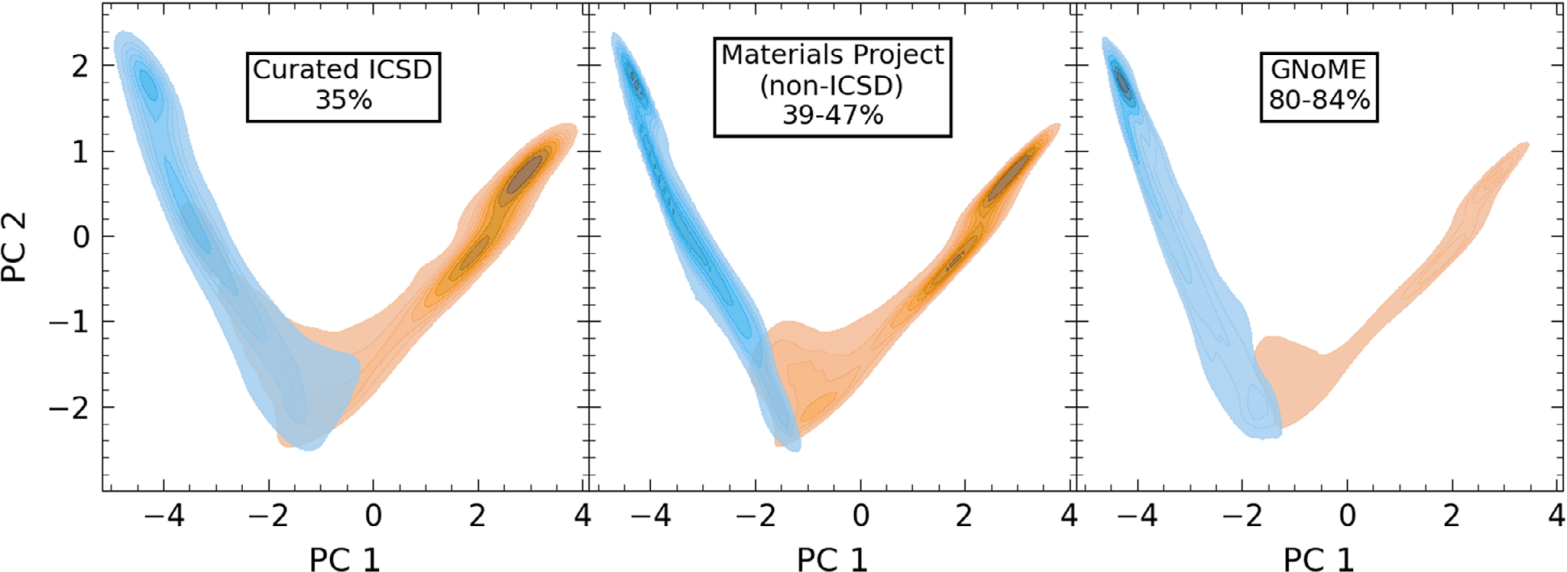
Principal component analysis of RNN pooled composition representations. Shown is a kernel density estimation of ordered (red) and disordered (blue) compositions of the curated ICSD (left), the Materials Project excluding materials also contained in the ICSD (center), and the GNoME discovery data set (right). The principal components are determined based on the RNN pooled composition representations for materials of the ICSD, while materials from MP and GNoME are projected into the space spanned by the ICSD principal components. We predict the rate of disorder in the database using the thresholds 50% (upper bound) and 70% (lower bound) and find values of 39–47% for MP and 80–84% for GNoME.

The differences between these data sets are further illustrated in **Figure** **3**, which displays the two principal components of the RNN latent space representations of compositions from the curated ICSD (left), MP (center), and GNoME (right). Here, the principal components are determined for the curated ICSD, while MP and GNoME compositions are projected into the same 2D space. A kernel density estimation (KDE) of the distribution of ordered (orange) and disordered materials (blue) illustrates that these are more or less cleanly separated by the first principal component of the latent space of the model. Notably, this is not the case when using a simple two‐dimensional embedding based on electronegativities and atomic radii (see Figure S7, Supporting Information). The curated ICSD and the MP display fairly similar distributions, consistent with their related origin and comparable degrees of disorder. For GNoME, the situation is drastically different with the highest probability density found in the disordered domain, at the boundary of the latent space.

The differences between these distributions are consistent with our hypothesis that a high degree of disorder in the GNoME convex hull is due to a bias in the investigated compositions. On the other hand, the disjoint nature of the respective sets of compositions also represents a sizable covariate shift with respect to the training set of the classifier, so that our predictions here are attached with a significant degree of uncertainty. Nevertheless, even the conservative estimate of 80% is much higher than the ICSD and MP values. Additionally, it should be emphasized that the classifier currently does not consider positional disorder, so that the overall degree of disorder may also be underestimated.

These findings illustrate that substitutional disorder is an important, underappreciated factor in computational materials discovery. Our classifiers predict a sizable fraction of disorder in MP and GNoME, with the latter potentially being dominated by disordered systems. This finding underlines that predictions presented in such efforts should always be taken with a grain of salt and ultimately require stronger experimental or computational evidence to back up claimed discoveries of novel, ordered phases. As a case in point, while some of the computationally predicted MP materials were experimentally validated with an autonomous synthesis setup,^[^ ^63^ ^]^ Leeman et al. subsequently found that known disordered phases exist for 23 of the 35 synthesized materials.^[^ ^29^ ^]^ For these, the balanced RNN model predicts that 16 (70%) are likely disordered. In the same way, Cheetham and Seshadri analyzed 10 random examples from the original GNoME database and found structural analogues in the ICSD that suggest artificial atomic ordering in all 10 computationally predicted structures.^[^ ^64^ ^]^ Again, the RNN model (with the balanced 50% classification threshold) predicts 9 of these 10 materials (90%) to be likely disordered, with the exception of Ac_4_P_8_S_2__8_Tl_8_. Actinoids are severely underrepresented in the ICSD however, so that this claim cannot be made with high confidence. These tests provide a limited validation of the model in an out‐of‐domain setting and indicate that it could be an important component in automated materials discovery (and synthesis) efforts.

## 3 Conclusion

This work investigates the importance of crystallographic disorder for computational materials discovery. Analyzing the ICSD,^[^ ^48^ ^]^ we demonstrate that disorder is a common phenomenon in real materials. We also find that disorder is closely linked to element similarity on the modified Pettifor scale, as also recently noted by Antypov et al.^[^ ^65^ ^]^ Substitutional disorder in particular follows distinct chemical patterns. Based on this insight, ML classifiers are developed that identify the occurrence of substitutional disorder from the composition alone with an accuracy of 90%. Applying these classification tools to large computational databases such as the Materials Project (MP) or GNoME,^[^ ^1^, ^4^ ^]^ we can estimate the prevalence of substitutional disorder in these materials spaces. In MP, the ML models predict that this is the case in 39–47% of systems, on a similar order to what is observed in the ICSD itself. In contrast, the more recent GNoME convex hull is estimated to display substitutional disorder in 80–84% of the cases.

This result aligns well with recent reports that 23 of the 35 synthesized materials from MP (that are also persistent on the GNoME convex hull) indeed have known disordered phases,^[^ ^29^, ^63^, ^66^ ^]^ as well as other reports of 10 random compositions from GNoME which have been suggested to be disordered phases.^[^ ^64^ ^]^ Another example is the case of TaCr_2_O_6_ proposed in the recent MatterGen effort,^[^ ^12^ ^]^ which is also predicted to be disordered by the RNN model (with the balanced 50% classification threshold). This compound was indeed found to reveal site disorder between Ta and Cr.^[^ ^12^, ^67^ ^]^ Similarly, Cerqueira et al. predicted LiMoN_2_ to be a promising candidate for a high‐temperature conventional superconductor in a high‐throughput screening, but subsequently found that the material displays positional disorder, which thwarts its beneficial properties.^[^ ^68^ ^]^ These examples underscore the importance of developing ML models that can predict the likelihood of disorder in computationally predicted materials.

It is important to note that our results should not be interpreted to mean that computational predictions of ordered crystals are not useful. To the contrary, databases like MP or GNoME are invaluable resources that have delivered important insights into the structure and properties of real and potential solid materials. Even when a material has a propensity for disorder, the ordered phase can be used to estimate its properties to some extent. Nevertheless, future research should explicitly account for the existence of crystallographic disorder in materials design. The classification models provided in this study can easily be integrated into computational or experimental discovery workflows. Another interesting approach that was presented very recently is Dis‐CSP, a generative model proposed by Petersen et al.,^[^ ^69^ ^]^ that is trained on the ICSD to specifically generate ordered and disordered crystal information files as appropriate, enabling the design of disordered materials, e.g., for novel disordered rock salt cathode (DRX) materials. However, both the current approach and Dis‐CSP are limited by the scarcity of data on disordered materials beyond the ICSD. Here, computational work (e.g. using Monte Carlo simulations and universal ML potentials) could play an important role to further improve the ML models presented here, essentially forming a closed loop between first‐principles modelling and ML prediction.

## 4 Experimental Section

### ICSD Preparation and Curation

The ICSD is accessed via the custom API provided by the FIZ Karlsruhe. We use the ICSDClient tool to query all 225 365 crystallographic information files (cif),^[^ ^70^ ^]^ and process them using ase and the cif parser pycodcif.^[^ ^71^, ^72^ ^]^ Skipping invalid elements such as M, X, or L, we arrive at 219 719 structures for 138 426 unique compositions.

These materials are then further curated to ensure a homogeneous data set for classification. First, we select only materials measured at normal temperature and pressure, i.e., a temperature of 293 K and a pressure of 101.325 kPa. This criterion is fairly restrictive and removes 70 432 entries from the data set. In addition, we limit the data set to substitutional disorder and remove materials with positional disorder. This is achieved by removing structures in which a crystallographic site is occupied by only one element and with an occupation <1. In this step, we remove another 30 603 entries, leaving us with the final data set containing 118 684 structures of 79 788 unique compositions. Of these structures, 77 571 (65%) are ordered and 41 113 (35%) are disordered. The entries in this curated data set are divided into a training, validation, and test split with a ratio of 60%: 10%: 30%. The full workflow is shown in the Supporting Information (Figure S3).

### Classification Models

The models presented in the following are implemented using pytorch. To generate the composition representations, we access element embeddings using the elementembeddings package.^[^ ^53^ ^]^ We optimize the ML models using the Adam optimizer and binary cross entropy loss. Hyperparameters are optimized with raytune by minimizing the loss on the validation set using a random search with 50 trials for each classifier and element embedding.^[^ ^73^ ^]^ The full search space of hyperparameters is shown in the Supporting Information alongside a number of performance metrics for the final classification model (see Tables S1–S3 and Figures S5 and S6). The optimal hyperparamters for each model are outlined in **Table** **1**. The code behind all models is available at: https://thgitlab.rz‐berlin.mpg.de/jakob/disorder‐classification.

**Table 1:** Set of optimized parameters determined with ray tune.

Parameter	Mean pooling	RNN pooling
Embedding	Mat2Vec^[^ ^57^ ^]^	Mat2Vec^[^ ^57^ ^]^
Scaled	False	False
*N*_hidden_	256	64
*N*_epochs_	400	300
Batch size	500	500
Learning rate	5 · 10^−4^	1 · 10^−3^

### Classification Models—Baseline Model

The baseline model uses the pair probability matrix shown in Figure 1 in order to classify compositions into ordered and disordered. Hence, we generate an identical matrix for the training set to avoid a prediction bias. Entries with no information are treated as 0, i.e., it is assumed that element pairs that have not been observed experimentally do not contribute to the prediction of disorder. For the prediction, we enumerate the element pairs present in each of the materials and evaluate the probability for these pairs. The maximum then determines the probability of disorder of the entire composition.

### Classification Models—Mean pooling model

For the mean pooling model, we generate element embeddings and perform a compositional average using the composition_featurizer feature of the aforementioned package. The embedding is treated as a hyperparameter and optimized as such. The composition embedding (i.e., the result of the pooling) is fed to a two‐layer multi‐layer perceptron (MLP) using a rectified linear unit activation between the layers. Finally, the output is processed with a sigmoid activation to obtain a probability of disorder.

### Classification Models—RNN pooling model

For the RNN model, we first sort elements in the composition by ascending electronegativity. Consequently, element tokens are converted into element embeddings and weighted with the composition‐normed molar fraction of the specific element. We pad the representation to a length of 16 elements and truncate compositions with more than 16 elements for practical reasons. The weighted element embeddings are then processed sequentially using a long short‐term memory (LSTM) unit as RNN. The last output of the LSTM is then fed through a two‐layer MLP readout as in case of the mean pooling model, combined with a rectified linear unit activation and finally a sigmoid activation to obtain a probability.

### Prediction of GNoME and Materials Project

The MP is accessed through the corresponding API.^[^ ^1^ ^]^ The version of MP used in this work contains 155 361 materials corresponding to 105 807 unique compositions. We remove compositional duplicates and predict disorder using the RNNs operated with two different thresholds of 50% and 70% for disorder. In this subset, we find 41 671 (39%) compositions with a matching entry in the ICSD (identified using the MP API search field database_IDs) and the slight majority of 64 136 (61%) compositions without.

In case of GNoME, we download the current version of the discovery data set available on GitHub (accessed on Feb, 2025).^[^ ^4^ ^]^ This data set comprises 554 054 structures within 1 meV of the convex hull, all of which correspond to unique compositions. We do not curate GNoME any further and generate the representation for the RNN to predict the disorder labels in the same way as for MP.

### Experimental Data Curation

In this work, we discuss combined experimental insights from refs. [63] and [29] using the ML classifiers. The respective data set was generated manually by collecting successful synthesis attempts from ref. [63]. These materials were labeled as ordered or disordered according to the evidence presented in ref. [29]. This means that compositions are labeled as disordered if no evidence supporting cation order was presented and a known disordered phase for the same composition exists. Out of the 36 materials marked as successful candidates, 35 were analyzed in ref. [29] and are thus analyzed herein. Note that also an unsuccessful synthesis attempt (Y_3_In_2_Ga_3_O_1__2_) was labeled as successfully synthesized and thus analyzed in ref. [29]. However, we omit this composition in our analysis.

## Conflict of Interest

The authors declare no conflict of interest.

## Supporting information

Supporting Information

## Data Availability

The data that support the findings of this study are openly available in MPCDF gitlab at, https://thgitlab.rz‐berlin.mpg.de/jakob/disorder‐classificationl , reference number 0.
