# Supplementary material for: Learning Crystallographic Disorder: Bridging Prediction and Experiment in Materials Discovery
Source: Adv Mater. 2025 Oct 23;38(5):e14226. doi: 10.1002/adma.202514226 (PMC12822528; doi:10.1002/adma.202514226)
Supplement: Supplementary file 1 — Supporting Information [file ADMA-38-e14226-s001.pdf]

ADVANCED MATERIALS

Supporting Information

for *Adv. Mater.*, DOI 10.1002/adma.202514226

Learning Crystallographic Disorder: Bridging Prediction and Experiment in Materials Discovery

*Konstantin S. Jakob, Aron Walsh, Karsten Reuter and Johannes T. Margraf**

Supplementary Information to: Learning Crystallographic Disorder: Bridging Prediction and Experiment in Materials Discovery

Konstantin S. Jakob, Aron Walsh, Karsten Reuter, Johannes T. Margraf

16.09.2025

S1 Disorder in the ICSD

During the data set curation of the inorganic crystal structure database (ICSD), we remove entries that were not measured at normal temperature and pressure (NTP) conditions, i.e. a temperature of 293 K and a pressure of 101.325 kPa. Figure S1 and Figure S2 show the distribution of measurement temperature and pressure for entries in the ICSD as well as the NTP conditions. The full workflow is shown in Figure S3.

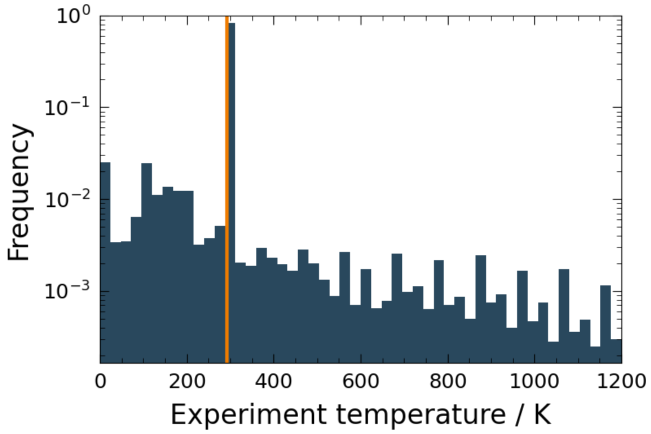

Supplementary Figure S1: Distribution of measurement temperature for materials in the ICSD.

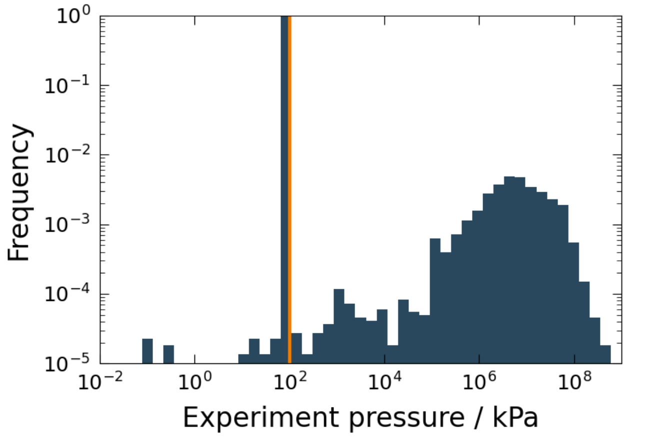

Supplementary Figure S2: Distribution of measurement pressure for materials in the ICSD.

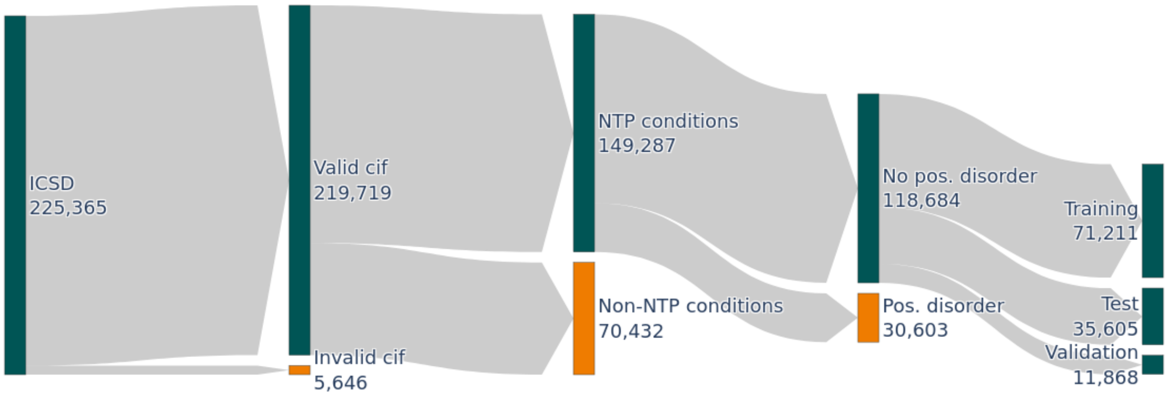

Supplementary Figure S3: Overview of the ICSD curation workflow. Sets denoted in orange were discarded in the process.

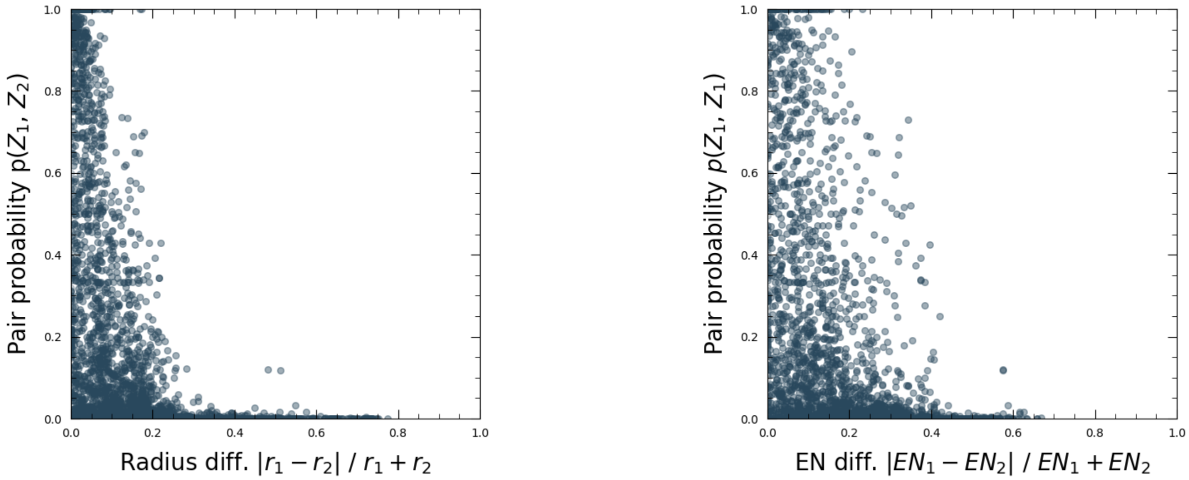

Supplementary Figure S4: Correlation of the element pair disorder probabilities presented in Figure 1C with differences in elemental properties such as covalent radius (left) or electronegativity (right).

S2 Classification Models

In addition to the classifier analysis in the main manuscript, we provide the learning curves (Figure S5) and confusion matrices (Figure S6) for the classification models.

Furthermore, the search space outlined in Table S1 was used in the hyperparameter optimization with `ray tune`. We train each model for 50 distinct sets of hyperparameters and perform a grid search over the element embedding, leading to a total of 150 trials. The mean pooling and RNN models with minimal validation loss across all epochs and hyperparameter combinations were used for the analysis in the main manuscript.

Supplementary Table S1: Hyperparameters and respective ranges for the classifier optimization with `ray tune`.

Hyperparameter	Mean pooling model range	RNN model range
Embedding	Matscholar, Mat2Vec or cgnf	Matscholar, Mat2Vec or cgnf
Scaled	True or False	True or False
N_{hidden}	$[32, 64, 128, 256]$	$[32, 64, 128, 256]$
N_{epochs}	$[50, 100, ..., 500]$	300
Batch size	$[50, 100, ..., 500]$	$[50, 100, ..., 500]$
Learning rate	$\left[1 \cdot 10^{-4}, 5 \cdot 10^{-4}, 1 \cdot 10^{-3}, ..., 1 \cdot 10^{-1}\right]$	$\left[1 \cdot 10^{-4}, 5 \cdot 10^{-4}, 1 \cdot 10^{-3}, ..., 1 \cdot 10^{-1}\right]$

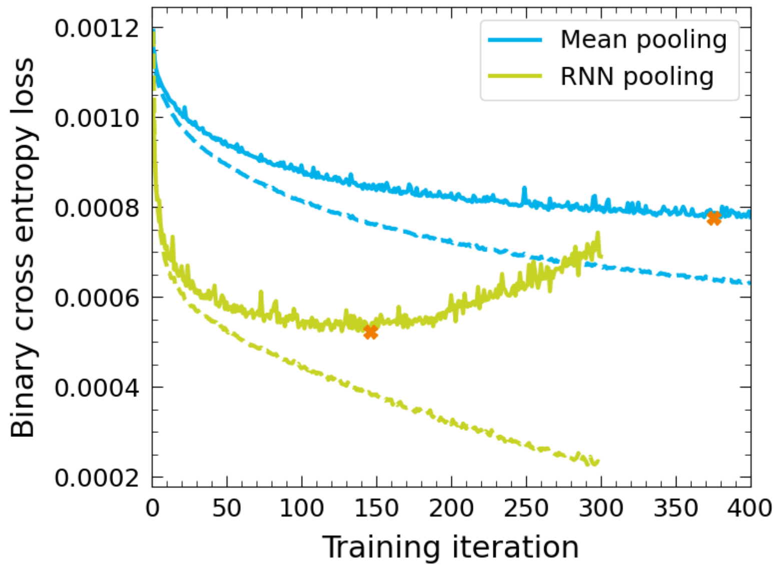

Supplementary Figure S5: Loss curves for the mean pooling model (blue) and the RNN model (green) with optimal hyperparameters evaluated on the training set (dashed) and the validation set (solid). The best model (orange cross) for each architecture is selected based on the epoch with minimal validation loss.

Finally, the RNN classifier achieves the performance metrics given in Table S2 and Table S3 with probability thresholds of 50% and 70%, respectively:

Supplementary Table S2: Performance metrics of the optimal RNN model with a disorder threshold of 50%.

Metric	Score (ordered)	Score (disordered)
Precision	92%	86%
Recall	93%	85%
f1-score	92%	86%
Support	23256	12349
Total accuracy		90%

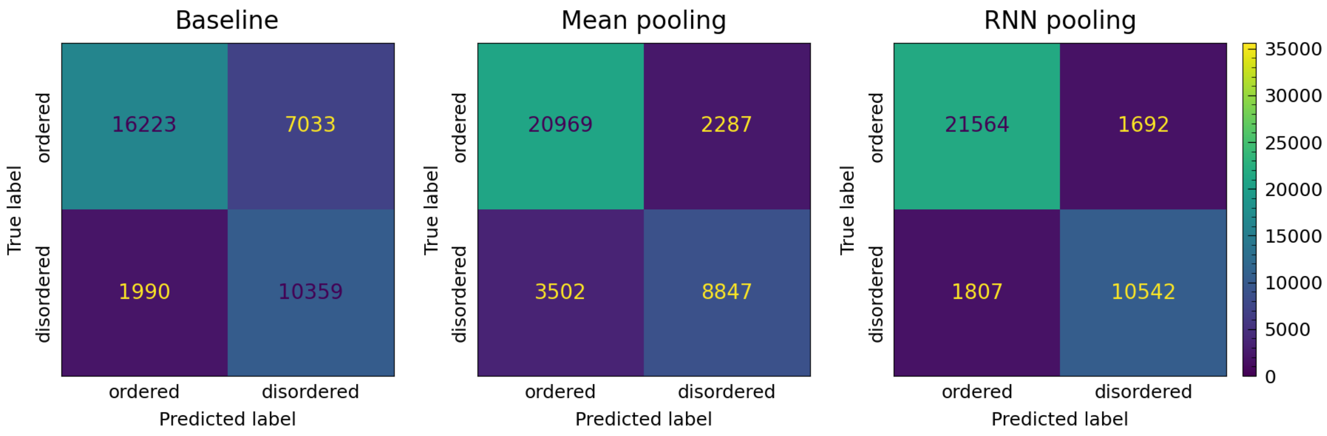

Supplementary Figure S6: Confusion matrices for baseline model (left), mean pooling model (middle), and RNN model (right) evaluated on the ICSD test set.

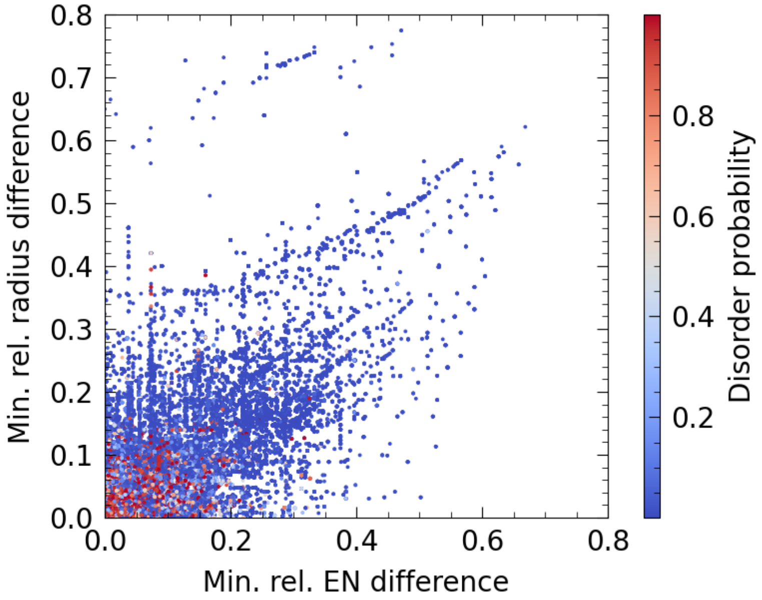

Supplementary Figure S7: Dependence of the RNN disorder probability on interpretable features such as the minimum, relative difference in covalent radii or electronegativity between all elements in the composition. The features are unable to clearly separate ordered and disordered compositions, unlike the principal components of the RNN representation.

Supplementary Table S3: Performance metrics of the optimal RNN model with a disorder threshold of 70%.

Metric	Score (ordered)	Score (disordered)
Precision	89%	90%
Recall	95%	78%
f1-score	92%	83%
Support	23256	12349
Total accuracy		89%

In addition to the composition-based models presented in this work, we train a joint RNN model on element embeddings and CrystalNN site fingerprints, a purely geometric descriptor representing the coordination environment of crystallographic sites. These representations are fed through the RNN simultaneously in separate channels for the most general description of a material that is possible in realistic materials discovery applications. Note that the crystal site representation must be agnostic to the occupying species, as computational predictions cannot incorporate partial occupancies. Further, global identifiers such as the space group cannot be used as the artificial ordering in computationally predicted structures can lead to symmetry lowering. The structure-aware model performs marginally better ($\sim$ 2%) than the best composition-based RNN. However, as the computational structure predictions carry some uncertainty due to artificial ordering effects, we focus on the purely compositon-based model in this work.

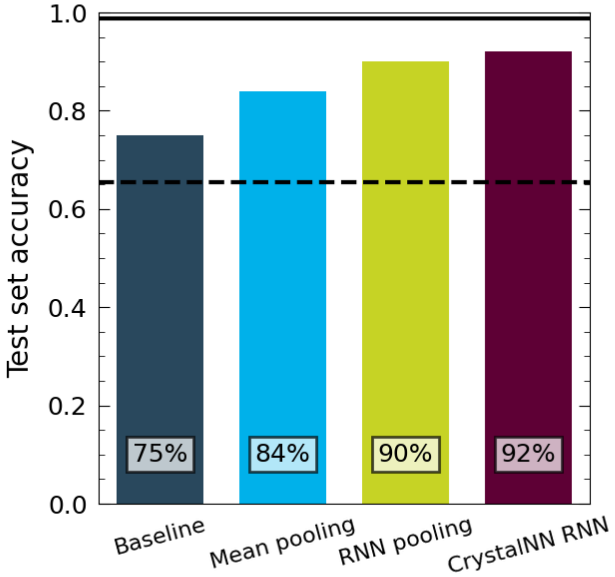

Supplementary Figure S8: Test set accuracy of the classifiers as shown in Figure 2 including an additional, structure-aware RNN classifier based on CrystalNN site fingerprints.
